# Hydrolytic Degradation Profile and RP-HPLC Estimation of Cilostazol in Tablet Dosage Form

**DOI:** 10.4103/0250-474X.41459

**Published:** 2008

**Authors:** P. K. Basniwal, P. K. Shrivastava, Deepti Jain

**Affiliations:** LBS College of Pharmacy, Tilak Nagar, Jaipur-302 004, India; 1 School of Pharmaceutical Sciences, Rajiv Gandhi Proudyogiki Vishwavidyalaya (University of Technology of MP), Airport Bypass Road, Gandhinagar, Bhopal-462 036, India

**Keywords:** Hydrolytic degradation, cilostazol, RP-HPLC

## Abstract

A simple, selective, precise and stability-indicating high-performance liquid-chromatographic method of analysis of cilostazol in pharmaceutical dosage form was developed and validated. The solvent system consisted of 10 mM phosphate buffer (pH 6.0):acetonitrile:methanol (20:40:40). Retention time of cilostazol in C18 column was 5.7 ± 0.1 min at the flow rate 1.3 ml/min. Cilostazol was detected at 248 nm at room temperature. The linear regression analysis data for the calibration plots showed good linear relationship with correlation coefficient value, r^ 2^ =0.9998 in the concentration range 100–3200 ng/ml with slope 43.45 intercept 156.75. The method was validated for linearity, range, accuracy, precision and specificity. Cilostazol was determined in tablet dosage form in range of 99.58-100.67% with 0.4600 standard deviation. Stress studies were conducted in acid and alkali hydrolysis with gradual increasing concentration. Cilostazol was found to be stable in various concentrations of acidic and alkaline.

A number of pharmaceutical substances have ester or amide as functional groups which may undergo hydrolysis in solutions or in aqueous suspension. Hydrolytic reactions involve nucleophilic attack on labile bonds such as lactam, ester, amide, imine and so on, by water on the drug in the solution and it follows first order kinetics^1^,^2^. Literature reveals that hydrolytic degradation is performed in neutral, acidic and alkaline conditions.

Cilostazol, chemically 6-[4-(1-cyclohexyl-*1H*-tetrazol-5-y1)-butoxy]-3,4-dihydro-2(*1H*)– quinolinone (fig. 1), is a quinolinone derivative that inhibits cellular phosphodiesterase III, and is used for inhibition of platelet aggregation and as a vasodilator 3–6. Literature survey reveals that only one chromatographic method is reported for quantitative analysis of cilostazol and its metabolites in human plasma^7^.

**Fig. 1:**
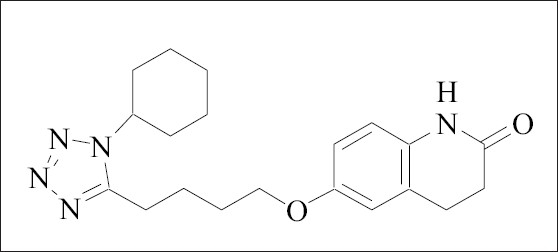
Structure of cilostazol.

In the present work, RP-HPLC method was developed and validated for quantitative estimation of cilostazol in tablet dosage form and hydrolytic degradation of cilostazol was performed and analysized by validated RP-HPLC method.

Cilostazol working standard was gifted by IPCA laboratories, Ratlam (MP) and solvents acetonitrile and methanol were HPLC grade from Merck Ltd., India. All other chemicals (sodium hydroxide, hydrochloric acid and potassium hydroxide) were analytical grade from Merck Ltd., India. Cilostazol tablets (Pletoz-50, Hetero Drugs Ltd., Hyderabad) were procured from local market.

For the RP-HPLC method development and hydrolytic degradation analysis of cilostazol instrument and separation variables are shown Table 1. Cilostazol shows retention time 5.7±0.1 min in the set of separation variables. Six replicates were injected separately to study system suitability parameters retention time (RT), area under curve (AUC), number of theoretical plates, tailing factors and height equivalent theoretical plates (HETP).

**TABLE 1:** INSTRUMENTS AND SEPARATION VARIABLES

Instrument	Parameters
HPLC system	
HPLC pump	LC-10ATvp Shimadzu
Column	Solvent delivery module LC-10ATvp Phenomenex (250 mm × 4.60 mm) Luna 5-4
Injector	C18(2),100A
Detector	Microliter syringes (Hamilton 702NR) SPD-M10 AVP-Shimadzu, UV/Vis Diode
Guard column	Array Detector
Operation software	Phenoxenex security Guard (universal fit) Class-LC10/M10A
Filter	Ufipore N_66_ Nylon 6, 6 membrane (pall life sciences)
Column	
Dimension	250 mm ×4.60 mm
Particle size	5 μm
Bonded phase	Octadecylsilane (C18)
Mobile phase	
10 mM phosphate buffer (pH 6.0)	20%
Acetonitrile	40%
Methanol	40%
Flow rate	1.3 ml/min
Temperature	Ambient
Sample size	20 μl
Detection wavelength	248 nm
Retention time	5.7±0.1 min

Accurately weighed about 100 mg cilostazol was dissolved in 50 ml methanol (HPLC grade) and volume was made upto 100 ml with triple distilled water (stock A, 1000 μg/ml). The stock solution was diluted to obtain 0, 100, 200, 400, 800, 1600 and 3200 ng/ml solution of cilostazol. The dilutions were fillered through 0.45 μm membrane filter and injected. Chromatograms were plotted and repeated for six times. A calibration graph was plotted between the peak AUC vs concentration and regression equation was AUC= 43.45X+156.75 with correlation coefficient r^2^ = 0.9998. The method was validated according to ICH guidelines^8^. RSD values of all validation parameters are far less than 2% (Table 2).

**TABLE 2:** VALIDATION PARAMETERS FOR CILOSTAZOL

Parameters	Values
Linearity	100-3200 ng/ml
Response ratio	43.63
SD of RR	0.1925
RSD of RR	0.0044
Range	200 - 1200 ng/ml
SD	16.54-37.10
RSD	0.0004 - 0.0023
Accuracy	
% Mean*	100.008
SD	0.065
RSD	0.0006
Precision	
Repeatability	
% Mean*	99.99
SD	0.1379
RSD	0.0014
Intermediate precision	
Day to Day	
% Mean*	100.03
SD	0.121
RSD	0.0012
Analyst to analyst	
% Mean*	100.03
SD	0.175
RSD	0.0017
Specificity	After hydrolytic degradation, peak response was same as previous because it is stable.

* - mean of six replicates, SD - Standard deviation, RSD - Relative standard deviation, RR - Response ratio, ng/ml - nanogram/milliliter

Twenty tablets (Pletoz-50, Hetero Drugs Ltd., Hyderabad) were weighed and finely powdered. Powder equivalent to 100 mg of cilostazol was dissolved in 50 ml methanol (HPLC grade) and volume was made upto 100 ml with triple distilled water. The sample was sonicated for 15 min and filtered through Whatmann paper no. 41 (stock P, 1000 μg/ml). 10 milliliters of this stock was diluted up to 100 ml with 50% aqueous methanol (stock Q, 100 μg/ml) and then further diluted up to 100 ml obtain stock R (10 μg/ml). Aliquots of 10 μg/ml were diluted to obtain concentration of 800 ng/ml and filtered through 0.45 μm membrane filter. Samples were analysed and statistical calculations were carried out (Table 3).

**TABLE 3:** ANALYSIS OF CILOSTAZOL TABLETS

Conc. of tablet dilution (ng/ml)	Area under curve	Concentration found (ng/ml)	% Found
800	35075	803.64	100.46
800	34825	797.89	99.74
800	35120	84.68	100.59
800	35012	802.92	100.37
800	34769	796.60	99.58
800	35150	805.37	100.67
Mean			100.24
SD			0.4600
RSD			0.0046
SEσ			0.1878

ng/ml - nanogram/milliliter, SD - Standard deviation, RSD - Relative standard deviation, SEσ - Standard error of standard deviation

Hydrolytic degradation in alkaline condition was carried out by dissolving accurately weighed 100 mg cilostazol in 50 ml methanol (HPLC grade) and volume was made upto 100 ml with 2 N NaOH. The solution was refluxed on water bath at 60º for 5 h. Aliquot of above solution was neutralized with 1 N HCl and diluted with diluent to obtain 800 ng/ml solutions. The sample solution was analysed and chromatogram was recorded. No degradation of cilostazol was found in 1 N NaOH at 60º after 5 h. Further, cilostazol was degraded in 2 N NaOH and 5 N NaOH and cilostazol was found to be stable.

Hydrolytic degradation under acidic conditions was performed by dissolving 100 mg cilostazol in 50 ml acetonitrile (HPLC grade) and volume was made upto 100 ml with 2 N HCl. The solution was refluxed on water bath at 60º for 5 h. Aliquots of above solution was neutralized with 1 N NaOH and diluted with diluent to obtain 800 ng/ml solutions. The sample solution was analysed and chromatogram was recorded. No degradation of cilostazol was found in 1 N HCl at 60º after 5 h. Further, the solution was degraded in 2 N HCl and 5 N HCl and was found to be stable.

Cilostazol tablets were analysed by validated RP-HPLC method and cilostazol was found in between 99.58-100.67% with relative standard deviation 0.0046. As cilostazol is insoluble in water and sodium hydroxide solution, cosolvent was required to perform alkali degradation of cilostazol. Acetonitrile as cosolvent was avoided for alkali degradation because it produces phase separation with 1 N or more concentrated NaOH solution^9^. So 50% methanolic sodium hydroxide solution was recommended to perform alkaline degradation of cilostazol. As per decision tree^10^ the degradation of cilostazol was performed in 50% methanolic 1 N, 2 N and 5 N NaOH at 60º for 5 h. Since there was no other peak (except cilostazol at RT 5.7±01 min) after treating by above stress conditions, cilostazol is stable drug under these conditions.

Cilostazol is also insoluble in hydrochloric acid and methanol as cosolvent is avoided with high concentration of HCl due to presence of amide group in cilostazol. Methanol may react with amide group and produce significant experimental artifact components^9^. The acidic degradation of cilostazol was performed in 50% acetonitrile 1 N, 2 N and 5 N HCl at 60º for 5 h and no peaks (except cilostazol at 5.7 ± 0.1 min) were seen after treating by above stress conditions. Thus, cilostazol is also stable in 50% acetonitrile HCl.

## References

[1] Wells JI (1988). Pharmaceutical preformulation: The physicochemical properties of drug substances.

[2] Walter L (1994). Pharmaceutical codes.

[3] Hashiguchi M, Ohna K, Nakasawa R, Kishino S, Mochizuki M, Shiga T (2004). Compression of cilostazol and ticlopidine for one-month effectiveness and safety after elective coronary stenting. Cardiovasc Drugs Ther.

[4] Sweetman SC (2002). Martinadale: The complete drug reference.

[5] Kishida M, Watanabe K, Tsuraoka T (2001). Effects of cilostazol in patients with bradycardiac arterial fibrillation. J Cardiol.

[6] Madias JE (2003). Cilostazol: An intermittent claudication remedy for management of third degree AV block. Chest.

[7] Bramer SL, Tata PN, Vengurlekar SS, Brisson JH (2001). Method for quantitative analysis of cilostazol and its metabilites in human plasma using LC/MS/MS. J Pharm Biomed Anat.

[8] (1996). International Conference on Hormonization Guidance for Industry Q2B Validation of Analytical Procdures. Methodology.

[9] Alsante MK, Friedmann RC, Hatajik TD, Lohr LL, Sharp TR, Synder KD, Ahuja S, Seypinski S (2001). Handbook of Modern Pharmaceutical Analysis.

[10] Singh S, Bakshi M (2000). Guidance on conduct of stress tests to determine inherent stability of drugs. Pharma Tech Online.

